# P-29. Impact of Reclassification of Serratia marcescens as a Low-Risk AmpC Pathogen on Treatment Selection and Outcomes in Bacteremia

**DOI:** 10.1093/ofid/ofaf695.258

**Published:** 2026-01-11

**Authors:** Jolie Schojbert, Dimple Patel, Aiman Bandali, Pamela Giordano, Robert Roland, Jason Kessler

**Affiliations:** Morristown Medical Center, Morristown, NJ; Atlantic Health System, Morristown, New Jersey; Atlantic Health System, Morristown, New Jersey; Atlantic Health System, Morristown, New Jersey; Overlook Medical Center, Summit, New Jersey; Morristown Medical Center, Morristown, NJ

## Abstract

**Background:**

In 2021, IDSA reclassified *S. marcescens* as a low-risk organism for clinically significant AmpC production, expanding treatment options to include ceftriaxone and piperacillin-tazobactam. In July 2021, the health system laboratory adopted the recommended reclassification, removed historic interpretive comments from the susceptibility report, and implemented selective reporting for cefepime and carbapenem susceptibilities. The objective of this study was to determine the impact of reclassification of *S. marcescens* as a low-risk AmpC pathogen on antibiotic selection and clinical outcomes.

**Figure:**
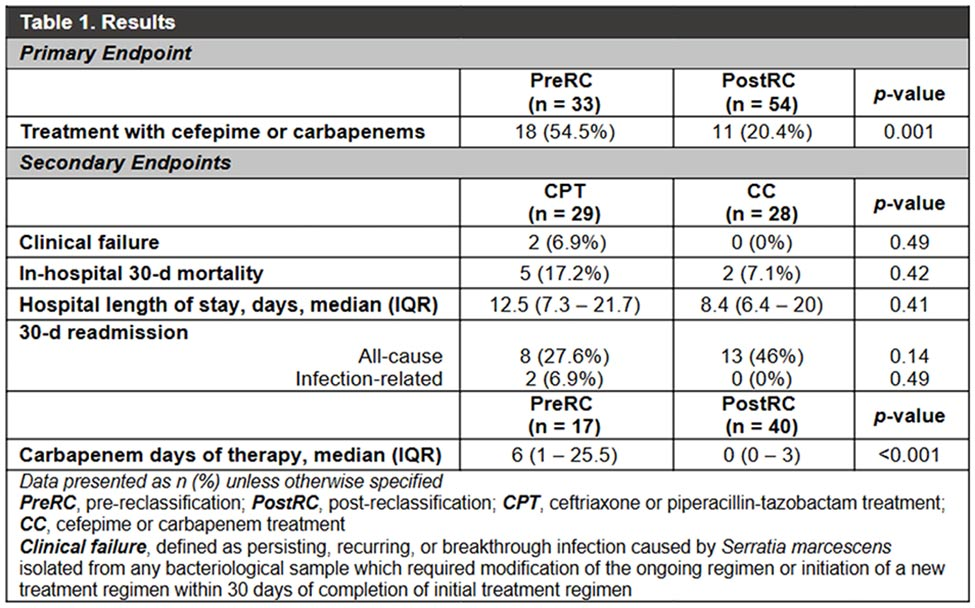

**Methods:**

This was a retrospective chart review of adult inpatients with *S. marcescens* bacteremia susceptible to ceftriaxone or piperacillin-tazobactam during the pre-reclassification (preRC; July 2018 – June 2021) and post-reclassification (postRC; July 2021 – December 2024) study periods. Patients were excluded if they were deceased or made comfort care prior to receiving definitive antibiotic therapy, discharged prior to susceptibility results, transferred from an outside hospital, neutropenic, had a presumed or documented concomitant infection or polymicrobial infection. The primary endpoint was the difference in percentage of patients treated with cefepime or carbapenems in both groups. Secondary endpoints were evaluated in the subgroup receiving ≥5 days of definitive monotherapy with ceftriaxone or piperacillin-tazobactam (CPT) or cefepime or a carbapenem (CC) within 48 hours of susceptibility results, and compared clinical outcomes based on treatment selection.

**Results:**

A total of 87 patients were included. An absolute reduction of 34.1% in treatment with cefepime or carbapenems was noted in the postRC group (54.5% vs 20.4%, p = 0.001). Secondary endpoints were analyzed in 57 patients. No statistically significant differences were noted in clinical failure, mortality, length of stay or readmission within 30 days. A significant reduction in median carbapenem days of therapy was observed in the postRC group (6 vs. 0, p<0.001) (Table 1).

**Conclusion:**

In patients with *S. marcescens* bacteremia, susceptibility reporting changes accompanying reclassification as a low-risk AmpC pathogen led to a reduction in treatment with cefepime and carbapenems.

**Disclosures:**

All Authors: No reported disclosures

